# Communication Interventions and Assessment of Drivers for Hendra Virus Vaccination Uptake

**DOI:** 10.3390/vaccines11050936

**Published:** 2023-05-04

**Authors:** Jessica N. Kropich-Grant, Kerrie E. Wiley, Jennifer Manyweathers, Kirrilly R. Thompson, Victoria J. Brookes

**Affiliations:** 1 Faculty of Science, Sydney School of Veterinary Science, The University of Sydney, Camperdown 2006, Australia; victoria.brookes@sydney.edu.au; 2 Sydney School of Public Health, The University of Sydney, Camperdown 2006, Australia; kerrie.wiley@sydney.edu.au; 3 Gulbali Institute, School of Agricultural, Environmental and Veterinary Sciences, Charles Sturt University, Locked Bag 588, Wagga Wagga 2678, Australia; jmanyweathers@csu.edu.au; 4 College of Health, Medicine and Well-Being, University of Newcastle, Callaghan 2308, Australia; kirrilly.thompson@newcastle.edu.au; 5 Hunter New England Local Health District, Wallsend 2287, Australia

**Keywords:** zoonoses, Hendra virus, vaccination, behaviour change, equine

## Abstract

Hendra virus disease (HeVD) is an emerging zoonosis in Australia, resulting from the transmission of Hendra virus (HeV) to horses from *Pteropus* bats. Vaccine uptake for horses is low despite the high case fatality rate of HeVD in both horses and people. We reviewed evidence-based communication interventions to promote and improve HeV vaccine uptake for horses by horse owners and conducted a preliminary evaluation of potential drivers for HeV vaccine uptake using the Behavioural and Social Drivers of Vaccination (BeSD) framework developed by the World Health Organization. Six records were eligible for review following a comprehensive search and review strategy of peer-reviewed literature, but evidence-based communication interventions to promote and improve HeV vaccine uptake for horses were lacking. An evaluation of potential drivers for HeV vaccine uptake using the BeSD framework indicated that horse owners’ perceptions, beliefs, social processes, and practical issues are similar to those experienced by parents making decisions about childhood vaccines, although the overall motivation to vaccinate is lower amongst horse owners. Some aspects of HeV vaccine uptake are not accounted for in the BeSD framework (for example, alternative mitigation strategies such as covered feeding stations or the zoonotic risk of HeV). Overall, problems associated with HeV vaccine uptake appear well-documented. We, therefore, propose to move from a problems-focused to a solutions-focused approach to reduce the risk of HeV for humans and horses. Following our findings, we suggest that the BeSD framework could be modified and used to develop and evaluate communication interventions to promote and improve HeV vaccine uptake by horse owners, which could have a global application to promote vaccine uptake for other zoonotic diseases in animals, such as rabies.

## 1. Introduction

Hendra virus (HeV; genus *Henipavirus*, family *Paramyxoviridae*) is an emerging zoonotic virus that has been detected in Australia’s four mainland species of *Pteropus* bats [1,2]. Transmission of HeV to horses is considered to be predominantly from *P. alecto* [2,3], and infected horses are the source of infection for people [1,4]. Since 1994, sixty-six spillover events between bats and horses have been recorded, resulting in 108 horse deaths and seven reported human infections, including four deaths [2,5,6]. There have also been two reported cases of HeV-infected dogs [7].

The geographical range of detected HeV disease (HeVD) in horses has expanded southward since its initial detection in Hendra, Queensland. Cases have recently been reported as far south as Scone [4,6] and West Wallsend, New South Wales, in 2019 and 2021, respectively [8]. The West Wallsend event was caused by a recently identified variant, HeV-g2 [2]. The variant has been detected in *P. poliocephalus* in South Australia, Victoria, Queensland, and New South Wales, and also in *P. alecto* in New South Wales [9,10].

Therapeutics for the treatment of HeVD are not available; however, a vaccine for horses and post-exposure prophylaxis for humans have been developed [1,2]. Horses aged ≥ 4 months can be vaccinated [11], with two doses given 3–6 weeks apart and a third dose six months later; then, an annual booster dose thereafter [11]. Despite the availability of the vaccine since 2012, the expanding range of detected HeVD events in horses, and the high case fatality rate of HeVD in both horses and humans, estimated HeV vaccine uptake ranges from 10.1% to 13.0% of the estimated horse population in Queensland and New South Wales annually from 2016 to 2021 [12]. As well as protecting equine welfare, HeV vaccination of horses remains a priority to protect public health [13,14].

The recent development of the Behavioural and Social Drivers of Vaccination (BeSD) framework has demonstrated value in understanding and promoting vaccine uptake by caregivers in the context of vaccine-preventable childhood diseases [15,16]. In this framework, there are four measurable domains of behavioural and social drivers in individuals that are potentially changeable: (1) *Thinking and Feeling*, relating to cognitive and emotional responses to vaccine-preventable diseases and vaccines, including disease risk perception and vaccine confidence; (2) *Social Processes*, defined as the social experiences related to vaccines, including gender equity, social norms about vaccination, and receiving health worker recommendations to be vaccinated; (3) *Motivation*, influenced by the former two domains and including vaccination intention, willingness, and hesitancy; and (4) *Practical Issues*, which are people’s experiences when trying to get vaccinated, including access barriers such as availability, affordability, ease of access, quality of service, and respect from health workers [16]. The combination of the four domains helps drive or hinder the uptake of recommended vaccines [16]. Research with non-vaccinating parents demonstrates a variety of reasons for childhood vaccine rejection, including low disease risk perception, fears about vaccine safety and efficacy, perceived “vested interests” of vaccine companies driving vaccine schedules, and perceived adverse events following previously administered vaccines [17]. Regardless of why they rejected vaccines, all parents’ decisions were underpinned by a desire to protect their children, and their decisions changed with time and circumstances [18]. Given the potential similarities between parents making vaccination decisions for their children and horse owners deciding on vaccinations for their horses, the BeSD framework may be useful in helping to understand the drivers of HeV vaccination.

The objective of this review was to identify peer-reviewed information about evidence-based communication interventions that have been used to promote HeV vaccine uptake by horse owners. We hypothesised that there are few interventions; therefore, a secondary objective was to appraise information in the reviewed records against the domains of the BeSD framework as a preliminary assessment of the types of information available about factors that drive or hinder HeV vaccine uptake. This review and assessment will inform research and development of a program to promote HeV vaccination.

## 2. Materials and Methods

### 2.1. Study Overview

The primary objective of the current study was to determine if there are evidence-based communication interventions to promote HeV vaccine uptake for horses. The secondary objective was to determine if the drivers of HeV vaccine uptake align with the domains of the behavioural and social drivers of vaccine uptake in the BeSD framework as a preliminary step to developing a framework for promoting HeV vaccine uptake.

To achieve the primary objective, we conducted a comprehensive review using methods for critically appraised topics [19,20] to identify descriptions and evaluations of evidence-based communication interventions to increase HeV vaccine uptake for horses by horse owners (see ‘Search strategy and review process’). To achieve the secondary objective, records identified as sufficiently relevant to Objective 1 to warrant full-text screening (Level 2; see ‘Search strategy and review process’) were assessed using the domains of behavioural and social drivers of vaccine uptake in the BeSD framework [15]. Ethical approval was not required for this review.

### 2.2. Definitions

The population of interest for the uptake of the HeV vaccine was people living in Australia who owned horses that could be at risk of HeVD. The term “communication intervention” was defined as any medium used to deliver information and ideas between parties to aid the discussion of topics of common interest, as well as inform, guide, and motivate a new or existing behaviour [21,22,23]. Communication interventions included face-to-face conversation, telephone conversation, videoconferencing, articles, pamphlets, email, blog posts, social media posts, website pages, smartphone apps, radio, and television [24]. The term ‘evidence-based’ in this context referred to interventions that were developed following research to systematically promote HeV vaccination uptake.

### 2.3. Search Strategy and Review Process

Searches were conducted in July 2022 using the terms “Hendra*” and “Vaccin*” and “Communicat*” or “Behavio* change” and “Horse owner*” and “Australia*” in BIOSIS Previews Via Web of Science, Scopus, and Medline via Ovid (Supplementary Information, Table S1).

Duplicates were removed, then records were screened for eligibility for Objective 1 (communication interventions to promote HeV vaccine uptake) based on the title and abstract (Level 1), then on the full record (Level 2). Records eligible for data extraction (Level 3) that identified communication interventions to promote HeV vaccine uptake were peer-reviewed and in English. Planned data extraction included the targeted population, type of communication intervention, including the method of dissemination or use, and pre- or post-use evaluation.

If records were not eligible for Level 3 data extraction for the primary objective but contained information about drivers of HeV vaccine uptake (secondary objective), data were extracted from each record and reported in a summary table including the population included in the study with their location and size, equine sectors represented, study design with objectives and factors studied, and main findings in the context of HeV vaccine uptake and the BeSD domains. The main findings were assessed for consistent and divergent (extraneous) themes according to the domains of the behavioural and social drivers of vaccine uptake in the BeSD framework (Thinking and Feeling, Social Processes, Practical Issues, and Motivation) and described below [15]. Extraneous themes included drivers of HeV vaccine uptake that extended beyond the domains of the BeSD framework.

## 3. Results

Thirty-three records were identified in the database searches, and six duplicate records were removed. Following a review of the title and abstract (Level 1), six records were eligible for review at Level 2. These records contained information from five studies (two records described different aspects of the same study) conducted from 2017 to 2021. The studies included a total of 239 participants (198 horse owners, 16 veterinary staff, and 25 members of the public).

**Figure 1:**
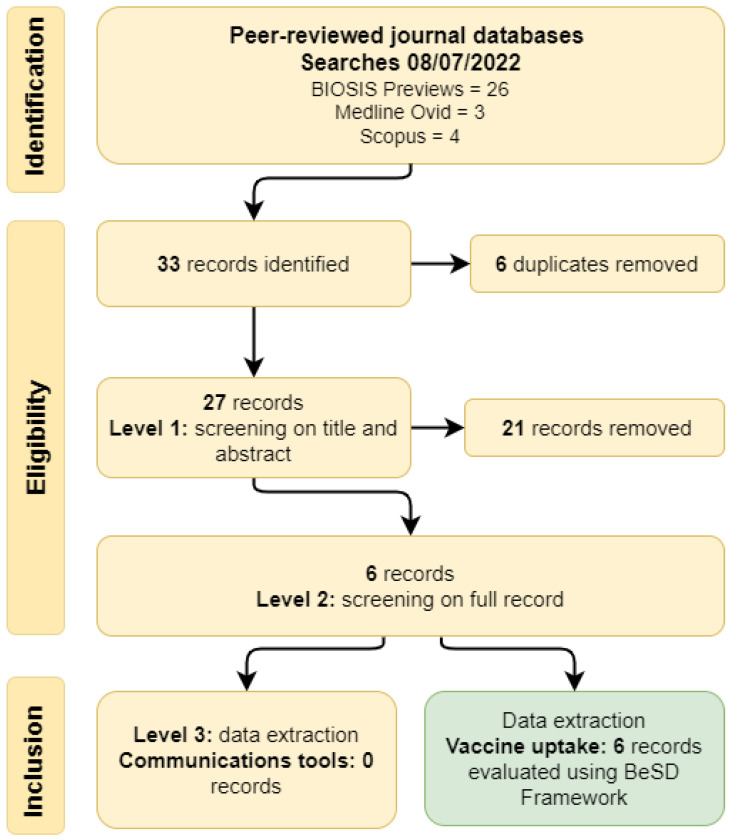
Review process and records identified in a systematic review to identify evidence-based communication interventions to promote vaccination against Hendra virus in horses and assessment of HeV vaccine uptake using the Behavioural and Social Drivers of Vaccination (BeSD) framework [16].

**Figure 2:**
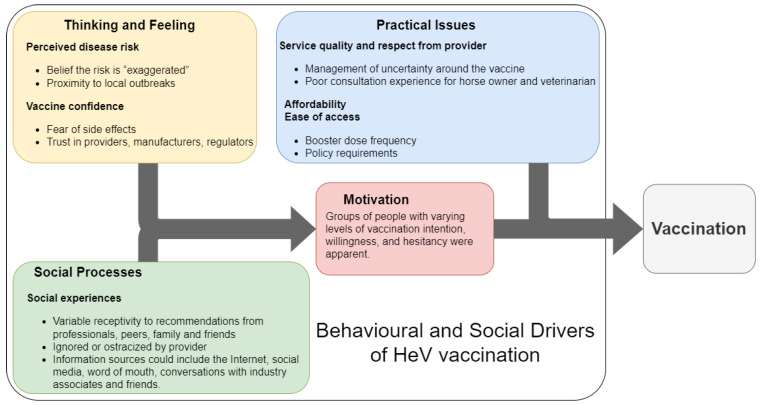
Diagram of the domains of the Behavioural and Social Drivers of Vaccination framework [16] and information obtained from records identified in a systematic review to identify evidence-based communication interventions to promote vaccination against Hendra virus infection in horses.

No records described evidence-based communication interventions to promote HeV vaccination uptake for horses (Figure 1: Level 3—data extraction, communications interventions). However, all of the records contained information that was illustrative of at least one of the four domains of the BeSD framework (Figure 1: Level 3—data extraction, vaccine uptake), as well as aspects that extended beyond the BeSD framework due to the One Health nature of HeV and the need to consider ecological drivers of disease spread and spread between populations (horses and people). This information is summarised in Table S2 and Figure 2 and described below.

### 3.1. Extracted Data—Drivers of HeV Vaccine Uptake

#### 3.1.1. Thinking and Feeling

Disease risk perception can be low, with some horse owners reporting that HeVD receives excessive attention and that the novelty of HeVD is exaggerated via scaremongering [25]. Risk perception can also change; the temporal and geographic proximity of HeVD outbreaks appears to trigger horse owners to seek information about HeVD and implement both pharmaceutical and property-focused HeVD risk mitigation strategies. Horse owners actively sought more information about HeVD following media reports of local outbreaks. The perceived immediacy of risk promoted HeV vaccine uptake [25,26,27].

Aspects of vaccine confidence, including perceived vaccine benefits and safety and trust in veterinarians and the authorities, were reported. Some owners had low vaccine confidence when they perceived a lack of HeV vaccine safety and efficacy or information transparency and availability [28]. These owners were more likely to discontinue HeV vaccination and believe in conspiracies between vaccine providers, manufacturers, and regulators, with a perception that horse and human welfare were not priorities for these groups [28]. Some owners requested HeV antibody titre testing as an alternative to HeV vaccination due to concern about HeV vaccine reactions and perceived over-vaccination [29]. In contrast, following a deliberative process, community juries who were comprised of the general public (not necessarily horse owners) were confident that vaccination mitigated HeVD risk safely and effectively, with some jurors agreeing that the promotion of HeV vaccination could be implemented immediately and affordably to increase vaccination uptake [14].

#### 3.1.2. Social Processes

Across the records, some social processes were reported as being influential in vaccination decision-making. Wiethoelter, Sawford, Schembri, Taylor, Dhand, Moloney, Wright, Kung, Field, and Toribio [25] reported that many horse owners sought opinions of veterinarians about HeVD and considered their veterinarians to be a valuable and trusted information source relative to governmental or social media sources. However, media reports could also trigger information seeking, and information sources were broad, including the Internet, social media, word of mouth, and conversations with contacts associated with horses and veterinarians. In a survey directed at horse owners who elected not to vaccinate their horses against HeV [26,27], most owners were not influenced by external social processes. Insurance and policy requirements (for example, veterinarians’ refusal to treat unvaccinated horses) and recommendations from veterinarians, medical doctors, industry peers, and friends were all unlikely to promote vaccine uptake. Horse owners could also feel ignored or ostracized following adversarial conversations with veterinarians, further undermining their trust in veterinarians, manufacturers, and regulators [28].

#### 3.1.3. Motivation

There was a range of motivational states described among participants in the included studies. Studies of horse owners who elected not to vaccinate horses demonstrated that there are groups with low motivation to vaccinate despite living in established HeVD risk zones [26,27,28]. These owners preferentially applied non-pharmaceutical and property-based mitigation strategies, such as covering food and water containers [27]. Other studies [25,29] described a range of levels of motivation to vaccinate among horse owners. A community jury study was conducted to assess the acceptability of adding ecological approaches to mitigate HeVD risk [14]. Following deliberation, jurors who reflected the public (and were not primarily horse owners) prioritised current interventions that included vaccination, as well as non-pharmaceutical and property-based mitigation strategies.

#### 3.1.4. Practical Issues

Findings related to the practical issues domain of the BeSD framework included service quality, respect from veterinarians, ease of access to the vaccine, and easily understood information, as well as cost and external requirements.

Service quality and respect were identified as important. For example, poor management of uncertainty around the HeV vaccine during a consultation or perceived ostracism by veterinarians led to a poor experience for both the horse owner and the attending veterinarian [28]. The understandability of the information being given was also found to be an important practical issue. Communication of test results and their meaning also presented challenges. In a study of the communication of HeV antibody titre results, veterinarians acknowledged the difficulty in communicating the results, a titre cut-off value, and the relationship between the titre value and protective immunity [29].

Access and affordability also influenced horse owners’ decisions about vaccine frequency in that the perceived high cost and need for ongoing boosters were barriers to HeV vaccination [26]. Other practical issues influencing the uptake of HeV vaccination were cost, booster dose frequency, policy requirements by veterinarians, and insurance requirements [22,23].

#### 3.1.5. One Health Aspects

Due to the complexity of HeV transmission and prevention, further issues were identified that extended beyond vaccine acceptance to include non-pharmaceutical approaches. These do not align neatly with the current vaccine-focused BeSD framework. This complexity was acknowledged in the community jury study in which participants identified the need for better communication and public education about the behaviours, ecological benefits, and zoonotic risks posed by bats [12]. For example, the proximity of outbreaks that increased disease risk perception and prompted horse owners to seek information was also likely to increase the uptake of property-focused HeVD risk mitigation practices, such as covering horses’ food and water containers, keeping horses off pasture when bats are active, and keeping horses away from fruiting/flowering trees [22].

## 4. Discussion

The comprehensive review of peer-reviewed literature did not identify any evidence-based communication interventions to promote HeV vaccination by horse owners. However, a subsequent assessment using the BeSD framework enabled the orientation and synthesis of research on horse owner HeV vaccine decision-making, suggesting that the BeSD framework could be adopted and modified to understand and promote HeV vaccination uptake. The BeSD framework was originally developed to characterise the drivers of parental decisions to vaccinate children that are individual, measurable, and potentially changeable with evidence-based interventions [15], and this review demonstrates that there are similarities between the underlying drivers of vaccination decisions among horse owners and parents. Most relate to the BeSD *Thinking and Feeling* domain in which non-vaccinating parents also perceive low disease risk, are concerned about vaccine side effects, believe that vaccines are unsafe and ineffective, and share a mistrust of health professionals [30,31,32]. Regarding the BeSD *Social Processes* domain, the social “othering” and dismissal by medical professionals that are experienced by some non-vaccinating parents [33] is comparable with the perceived ostracism from veterinarians reported by HeV vaccine-sceptical horse owners [28]. Within the same domain, healthcare provider recommendations to parents are akin to those from veterinarians to horse owners [17,30,34]. Within the BeSD *Practical Issues* domain, the cost of vaccination as an influence on horse owner vaccination decisions also influences parents’ vaccination decisions; for example, cost is a known barrier to the Meningococcal B vaccination, which is not included in the National Immunisation Program schedule for all non-Indigenous Australian children [35,36].

The BeSD *Motivation* domain is a point at which the parents and horse owners might differ. It is not possible to quantify the probability of horse owners’ intention to vaccinate (motivation) in the current review. However, it is clear that there are groups of horse owners with little motivation to vaccinate their horses against HeVD, and overall, motivation appears lower than that of parents for childhood vaccines because there is currently > 95% *versus* ~12% uptake for childhood vaccines and the HeV vaccine, respectively [33,37]. Generally, horse owners behave similarly to parents in that increased risk perception will result in increased vaccination motivation [17], but the complexity of HeV transmission necessitates that HeVD prevention extends beyond vaccine acceptance to include non-pharmaceutical approaches. The BeSD framework was designed for childhood and COVID vaccination, grounded in the evidence for the drivers of vaccination behaviour. Additional non-pharmaceutical behaviours for HeVD prevention may have different drivers that do not necessarily align well with the current vaccine-focused BeSD framework. This complexity was acknowledged in the community jury study, in which participants identified the need for better communication and public education about the behaviours, ecological benefits, and the zoonotic risk posed by bats [14]. Where the biggest barriers to HeV vaccination lie—whether associated with the domains of ‘Thinking and Feeling’ and ‘Social Processes’ or the domain of ‘Practical Issues’—needs further investigation beyond this preliminary appraisal.

The lack of vaccine uptake for HeV in Australia is paralleled in the United States (US), where there is similarly poor rabies vaccine uptake for horses. Rabies (genus *Lyssavirus*, family *Rhabdoviridae*) is caused by a fatal zoonotic neurotropic virus that is transmitted to horses by direct contact (bites) from infected bats, racoons, foxes, and skunks [38]. Similar to HeVD, rabies has a low incidence in horses with severe consequences in both humans and horses [29,39,40]. Thirteen cases of rabid horses and donkeys in the US were reported in 2018 [41]. Like HeV-infected horses, rabid equids can be a source of infection for people [42]. Whilst vaccination of horses against rabies virus infection is not mandatory in the US, the American Association of Equine Practitioners considers the rabies virus vaccine amongst the core vaccines and strongly recommends the annual vaccination of all equids against rabies [43]. In a 2017 study, it was reported that only one in seven horses are vaccinated against rabies in the US, equating to approximately 14% of the 7.25 million owned horses in the US [44,45]. The reasons for the poor uptake of the rabies vaccine by US horse owners and the HeV vaccine by Australian horse owners are similar. Many US owners report not vaccinating their equids (inclusive of domestic horses, miniature horses, ponies, mules, donkeys/burros, and zebra–donkey crosses) against specific diseases (including rabies) due to low exposure risk, effort, and the cost outweighing the benefit [46,47].

Considering the many parallels between non-vaccinating horse owners and non-vaccinating parents and the potential global public health impact, as well as the benefits to horse health and welfare, we are prompted to consider the applicability of communication tools that are available to medical professionals in approaching vaccine discourse with vaccine-hesitant and refusing parents. A clinical communication support intervention for complex communication around childhood vaccination has been developed by the Sharing Knowledge About Immunisation (SKAI) project [48]. The SKAI system has an accessible information website for parents and another for health professionals, including an eLearning module that is designed to adapt the clinical communication skills of health professionals to different immunization consultations [49,50,51]. The interface for parents provides information and resources tailored to address concerns for the level of intention, hesitancy, or refusal present [48,49,51]. The health professional interface similarly provides healthcare workers with tailored information and decision support tools for use before, during, and after immunisation consultations to address concerns and meet communication needs for the level of intention, hesitancy, or refusal present [48]. Research to better understand HeV or rabies vaccination drivers would underpin a modified equine BeSD framework and survey tool and inform the development of an HeV- or rabies-specific SKAI-type system to promote vaccination for these zoonoses in animals [48]. The implementation of such an intervention could then be evaluated using the BeSD tools to allow a comparison of the most effective communication tool to increase HeV vaccine uptake for horses by Australian horse owners.

## 5. Conclusions

Despite the health impact of Hendra virus disease (HeVD) on horses and people, there are no evidence-based communication interventions to promote Hendra virus (HeV) vaccine uptake for horses in Australia. Using the World Health Organization’s Behavioural and Social Drivers of Vaccination (BeSD) framework for childhood vaccine uptake, we highlighted both similarities and differences in the perceptions, feelings, motivations, social processes, and practical issues that horse owners and parents face in the context of HeV and childhood vaccine decision-making, respectively. This provides some insight into horse owners’ HeVD preventive behaviour and alludes to the circumstances in which they might consider HeV vaccination. This current understanding of the *why* behind the low uptake of HeVD risk mitigation behaviour by horse owners now needs to be understood in greater detail using mixed social research methods, including other influencing factors, such as the use of alternative mitigation strategies and zoonotic transmission risk, and how these preventive behaviours differ between horse owner types (e.g., recreational versus professional) [15,25,26]. A modified BeSD framework could be developed to produce interventions that reduce HeV vaccine coverage gaps [15], thus reducing the risk of HeVD to horses and, therefore, people. If successful, such modifications to the BeSD framework could have a further application to other zoonoses in One Health contexts, such as vaccination against rabies in endemic regions globally.

## Data Availability

No new data were created or analysed in this study. Data sharing is not applicable to this article.

## Supplementary Materials

The following supporting information can be downloaded at: https://www.mdpi.com/article/10.3390/vaccines11050936/s1; Table S1: Search strategy; Table S2: Summaries of the evidence.
