# Supplementary material for: Communication Interventions and Assessment of Drivers for Hendra Virus Vaccination Uptake
Source: Vaccines (Basel). 2023 May 4;11(5):936. doi: 10.3390/vaccines11050936 (PMC10221404; doi:10.3390/vaccines11050936)
Supplement: Supplementary file 1 [file vaccines-11-00936-s001.zip › vaccines-2324022-supplementary.pdf]

vaccines

MDPI

Supporting Information

Table S1. Search Strategy.

Database	Date Range	Search Terms
BIOSIS Previews Via Web of Science https://clarivate.com/webofsciencegroup/solutions/webofscience-biosis-previews/	1926-present	Hendra* (All Fields) and Vaccin* (All Fields) and Communicat* (All Fields) or Behavio* change (All Fields) and Horse owner* (All Fields) and Australia* (All Fields)
Scopus https://www.scopus.com/	1788-present	(TITLE-ABS-KEY (hendra*) AND TITLE-ABS-KEY (vaccin*) AND TITLE-ABS-KEY (communicat*) OR TITLE-ABS-KEY (behavio* AND change) AND TITLE-ABS-KEY (horse AND owner*) AND TITLE-ABS-KEY (australia*))
Medline via Ovid https://www.wolterskluwer.com/en/solutions/ovid/ovid-medline-901	1946 present	Hendra*.mp. and Vaccin*.mp. and Communicat*.mp. or Behavio* change.mp. and Horse owner*.mp. and Australia*.mp.

Table S2. Summaries of the evidence.

**"We've learned to live with it"—A qualitative study of Australian horse owners' attitudes, perceptions and practices in response to Hendra virus**<br>Wiethoelter, AK, Sawford, K, Schembri, N, Taylor, MR, Dhand, NK, Moloney, B, Wright, T, Kung, N, Field, HE, Toribio, J-A LML<br>Preventive veterinary medicine, 2017	
Population	Horse owners and/or care givers ≥18 years old from one of two 'hot spot' locations where equine and/or human HeVD cases had historically occurred (Northern NSW and central QLD). In the NSW location, the latest equine HeVD case was a single event that occurred in 2015. The latest HeVD cases occurring in the QLD location was of four equines in 2012.
Equine sectors represented	Riding associations i.e., Riding for the Disabled, Pony Club<br>Leisure/recreational<br>Work/farm i.e., stock work on cattle stations<br>Thoroughbred racing<br>Competition i.e., eventing, dressage, show jumping, campdrafting, endurance, polo, or polocrosse<br>Boarding facilities<br>Stud farms and horse breeders<br>Commercial enterprises i.e., riding schools, horse equipment stores, veterinary/equine health services
Data collection period	September - October 2014, i.e., prior to full APVMA registration of Equivac® HeV in August 2015 and class-action against Zoetis Australia PTY LTD in March 2018
Sample size	N = 27
Study type	Qualitative study
Data collection tool	A total of 24 face-to-face, in-depth, semi-structured interviews.<br>Each interview consisted of nine predominantly open-ended key questions covering 3 major topics: (1) experiences with HeV, (2) perception of HeV, and (3) communication around HeV.

Analytic approach	Interview audio recordings were transcribed for theoretical thematic analysis.
Objectives	To investigate self-reported experiences, attitudes, perceptions, and practices of horse owners in response to HeV.<br>To gain a deeper understanding of horse owners' decision-making around HeVD prevention measures.
Factors studied	Experiences, knowledge, attitudes, and practices in the context of HeV.
Main findings	Some horse owners believed that HeVD receives too much attention and that reports of the novelty of HeVD is exaggerated and scaremongering.<br>Media reports of local outbreaks of HeVD increased awareness and knowledge, and often functioned as triggers for horse owners to actively seek more information about HeV.<br>Sources of HeV information included the Internet, social media, word of mouth, conversations with contacts associated with horses, and veterinarians.
BeSD domains covered	Thinking and Feeling<br>Social Processes<br>Motivation
Considerations in the context of the current study	This study does not make comparisons between types of communication material that influence horse owners' vaccine uptake behaviour. It provides descriptive evidence about effects of information and types of information sources.

**"Why won't they just vaccinate?" Horse owner risk perception and uptake of the Hendra virus vaccine**<br>Manyweathers, J, Field, H, Longnecker, N, Agho, K, Smith, C, Taylor, M.<br>BMC veterinary research, 2017	
Population	Horse owners who had elected not to vaccinate their horses against HeV and were living geographically close to where HeVD cases had previously occurred (far north NSW, and South-East, Central, and Far North QLD). In far north NSW, the most recent equine HeVD case occurred in 2014. The most recent HeVD equine cases occurring in the QLD locations were in 2014 in both South-East and Central QLD, and 2013 in far north QLD (https://www.business.qld.gov.au/industries/service-industries-professionals/service-industries/veterinary-surgeons/guidelines-hendra/incident-summary, accessed 19 December 2022).
Equine sectors represented	Competitive/equestrian<br>Recreational<br>Working/farming/stock horse
Data collection period	Between January to March 2015 i.e., pre full APVMA registration of Equivac® HeV in August 2015 and pre class action against Zoetis Australia PTY LTD in March 2018
Sample size	N = 150
Study type	Cross-sectional survey and qualitative study [this paper is a part of the same study as the Manyweathers, Field, Jordan, et al. (2017) paper]
Data collection tool	Participants responded to an online survey disseminated via veterinary hospital Facebook pages and cross-postings to groups associated with the horse industry (e.g. dressage clubs). The survey consisted of 38 open and closed questions exploring horse owners' risk mitigation practices, risk perception, and attitudes to HeVD risk.
Analytic approach	Simple descriptive statistics, descriptive analysis, and theoretical thematic analysis.
Objectives	Identify factors associated with the uptake of the protective strategies to reduce the risk of HeV infection.<br>Determine how to improve uptake of HeVD management strategies.
Factors studied	Experiences, knowledge, attitudes, and practices in the context of HeV.
Main findings	Potential vaccination uptake enablers:<br>Reduction, elimination, or subsidisation of vaccine cost<br>Reduced booster dose frequency<br>Immediacy of HeVD risk<br>Administration of equine HeV vaccine by horse owner

	Policy (veterinary hospitals refusing to attend unvaccinated horses), insurance requirements, and recommendations by salaried experts (scientists, researchers, and risk analysts), others in the horse industry, or friends, were less influential
BeSD domains covered	Thinking and Feeling<br>Social Processes<br>Motivation<br>Practical Issues
Considerations in the context of the current study	Comparison of reasons for vaccination were not possible due to selection of horse owners who elected not to vaccinate their horses against HeV.<br>Although this study identifies circumstances in which non-vaccinating horse owners would reconsider vaccinating their horses, it does not identify nor compare types of communication tools that influence horse owner vaccine uptake.

**Risk mitigation of emerging zoonoses: Hendra virus and non-vaccinating horse owners**<br>Manyweathers, J, Field, H, Jordan, D, Longnecker, N, Agho, K, Smith, C, Taylor, M.<br>Transboundary and Emerging Diseases, 2017	
Population	Horse owners who were non- or partially vaccinating their horses against HeV and were living geographically close to locations where HeVD cases had previously occurred (far north NSW, and South-East, Central, and Far North QLD). In far north NSW, the most recent equine HeVD case occurred in 2014. The most recent HeVD equine cases occurring in the QLD locations were in 2014 in both South-East and Central QLD, and 2013 in far north QLD (https://www.business.qld.gov.au/industries/service-industries-professionals/service-industries/veterinary-surgeons/guidelines-hendra/incident-summary, accessed 19 December 2022).
Equine sectors represented	Competitive/equestrian<br>Recreational<br>Working/farming/stock horse
Data collection period	Between January to March 2015 i.e., pre full APVMA registration of Equivac® HeV in August 2015 and pre class action against Zoetis Australia PTY LTD in March 2018
Sample size	N = 150
Study type	Cross-sectional survey [this paper is a part of the same study as the Manyweathers, Field, Longnecker, et al. (2017) paper]
Data collection tool	Online survey disseminated via veterinary hospital Facebook pages.<br>The survey comprised of 38 open and closed questions exploring horse owners' risk mitigation practices, risk perception, and attitudes to HeVD risk.
Analytic approach	Simple descriptive statistics, descriptive analysis, and univariate and multivariate binary logistic regression analysis.
Objectives	Investigate the uptake of, and barriers to, the adoption of 4 property-focused HeVD risk mitigation practices/behaviours (covering horses' food and water containers, keeping horses off pasture when flying foxes are active, and keeping horses away from fruiting/flowering trees) amongst non- and partially vaccinating horse owners.<br>Identify additional pathways and reliable influencing factors to inform government policy to promote HeVD risk reduction by assisting stakeholders to engage and communicate with horse owners.
Factors studied	Experiences, knowledge, attitudes, and practices/behaviours in the context of HeV.
Main findings	Factors influencing the potential uptake of property-focused HeVD risk mitigation practices:<br>Actual or nearby HeV infection was most likely to influence uptake.<br>Government funding assistance could also contribute to uptake.

	Whilst less likely, professional (veterinarian or a consultant) support of property-focused HeVD risk mitigation was more likely to influence uptake than that of respected others or friends.
BeSD domains covered	Thinking and Feeling<br>Social processes<br>Motivation<br>Practical Issues
Considerations in the context of the current study	Absence of sample size determination and justification.<br>The group of participants were non-representative of all horse owners in Australia.<br>Comparison of reasons for vaccination were not possible due to selection of horse owners who elected not to vaccinate or partially vaccinate their horses against HeV.<br>This study does not identify and compare communication types that influence vaccine uptake behaviour by horse owners. Instead, it suggests positive influence of communication from veterinarians on the uptake of property management practices to mitigate HeVD risk by horse owners.

**Managing the risk of Hendra virus spillover in Australia using ecological approaches: A report on three community juries**<br>**Degeling, C, Gilbert, GL, Annand, E, Taylor, M, Walsh, MG, Ward, MP, Wilson, A, Johnson, J**	
PLoS One, 2018	
Population	Male and female residents of Rockhampton QLD, Lismore NSW, and Sydney NSW, who were ≥18 years old, with different levels of educational attainment, socio-economic status, and experience with horses. The latest HeVD equine case to occur in Rockhampton was in 2012, and in Lismore it was in 2017. There have been no HeVD cases reported in Sydney, however, at the time the closest equine HeVD case occurred in Casino NSW in 2016.
Equine sectors represented	Not reported
Data collection period	Between October 2017 and March 2018 i.e., post full APVMA registration of Equivac® HeV in August 2015 and at the onset of the class action against Zoetis Australia PTY LTD in March 2018
Sample size	N = 31
Study type	Qualitative study
Data collection tool	Three community juries (Rockhampton, Lismore, and Sydney) were each convened over two days to gain evidence about the views of well-informed citizens (horse owners, those with horse experience but not owners, and non-horse owners) on the appropriateness and perceived legitimacy of adding ecological approaches to current interventions that mitigate HeVD risk (horse vaccination and horse husbandry practices).<br>On day 1 of deliberation, each jury received extensive information from 4 experts about HeV and had the opportunity to ask the experts questions or clarify information. On day 2 of deliberation, each jury then reflected on, discussed, and debated the evidence, before deliberating, and reaching a final verdict on the questions for consideration. The verdicts, underpinning reasonings, and dissenting views were then reported to the research team.
Analytic approach	Audio recordings of each jury's deliberations and expert question and answer sessions were transcribed. The transcripts were subsequently reviewed and the key reasons for support or rejection of the questions for consideration in the jurors' own words were reported.
Objectives	Elicit, gain, and understand the views of well-informed citizens regarding the acceptability and perceived legitimacy of adding ecological approaches to current interventions that mitigate HeV spillover.
Factors studied	Opinions on the responsibilities of different stakeholders, current risk mitigation strategies, and the use of ecological approaches for risk mitigation in the context of HeV.

Main findings	All community juries ranked further promotion of equine HeV vaccination and HeV-safe horse husbandry practices as the highest priority approach to mitigate HeVD risk.<br>All community juries emphasized the need for better communication and public education about the behaviours, ecological benefits, and zoonotic risk that flying foxes pose.<br>Most jurors acknowledged that relying on horse owners to voluntarily vaccinate against HeV is an ineffective stand-alone strategy to minimise HeVD risk.<br>Some jurors agreed that the promotion of HeV vaccination could be implemented immediately and relatively cheaply to achieve increased vaccination uptake.
BeSD domains covered	Thinking and Feeling
Considerations in the context of the current study	This study investigates population-level approaches rather than individual-level approaches to HeVD risk mitigation.<br>This study does not investigate nor compare types of communication tools that influence vaccine uptake behaviour by horse owners. It provides information on the prioritisation of, challenges to, and suggestions for, vaccination and horse husbandry practices to mitigate HeVD risk by informed citizens.

**Expertise and communicating about infectious disease: A case study of uncertainty and rejection of local knowledge in discourse of experts and decision makers**<br>Manyweathers, J, Taylor, M, Longnecker, N<br>Journal of Science Communication, 2020	
Population	Horse owners that were non- or partially vaccinating their horses against HeV, and veterinary staff (veterinarians, nurses, and pathologists) practicing in locations known to be at risk of HeV (far north NSW, and South-East, Central, and Far North QLD).
Equine sectors represented	Competition/equestrian<br>Commercial enterprise i.e., veterinary/equine health services
Data collection period	July 2015 i.e., pre full APVMA registration of Equivac® HeV in August 2015 and pre class action against Zoetis Australia PTY LTD in March 2018
Sample size	N = 25
Study type	Qualitative study
Data collection tool	A total of 25, in-depth, semi-structured (23 face-to-face and 2 telephone) interviews were conducted.<br>A subset of horse owners (N = 15) was recruited via an online survey disseminated via veterinary hospital Facebook pages between January and March 2015. It is unclear how veterinary staff (N = 10) were recruited for this study.<br>Each horse owner interview consisted of 10 predominantly open-ended key questions covering 4 major topics: (1) general horse ownership, (2) horse health, (3) impact of HeVD and the vaccine, and (4) perceptions of other horse owners' vaccination decisions.<br>Each veterinary staff interview consisted of 10 predominantly open-ended key questions covering 4 major topics: (1) personal involvement with horses, (2) professional involvement with horses and horse owners, (3) impact of HeVD and the vaccine, and (4) attitudes towards horse owners in the context of HeV.
Analytic approach	Digital recordings of the interviews were transcribed for inductive thematic analysis.
Objectives	Explore the perspectives of those considered to be non-compliant with HeVD risk mitigation recommendations and the experts responsible for providing information on HeV and the vaccine.<br>Inform future risk communication strategies and management of emerging and re-emerging infectious diseases.
Factors studied	Experiences, knowledge, and attitudes in the context of HeV.
Main findings	Management of uncertainty and its effects on trust:

	A significant contributor to divisive communication between veterinarians and horse owners is the management of uncertainty of the HeV vaccine.<br>Horse owners desire evidence of:<br>HeV vaccine safety and efficacy,<br>Transparency of data, and<br>Openness around the information available to decision-makers of risk events.<br>A perceived lack of this evidence led to:<br>Discontinuation of the HeV vaccine protocol,<br>A sense that horse and human welfare were not a priority,<br>Increasing fear of the HeV vaccine, and<br>The belief of a conspiracy between veterinarians, the pharmaceutical company, the veterinary body, and the vaccine registration body.<br>Trust in veterinarians and uncertainty around HeV vaccination can co-exist with collaborative discourse.<br>A deficit approach to uncertainty and communication by veterinarians seemed to create more division in communication between veterinarians and horse owners.<br>How perception of expertise influenced inclusion in discussion:<br>Horse owners experiencing or knowing of HeV vaccine side-effects felt that they were being ignored in discussions around HeVD which further undermined their trust in veterinarians, the pharmaceutical company, and the vaccine registration body.<br>The ostracism by veterinarians that some horse owners experienced after raising concerns about HeV vaccine side-effects lead to fear to vaccinate again or to vaccinate new horses.<br>When local knowledge and experience was not included in communication around HeVD it resulted in failure of participatory communication, and damaged trust in the vaccine and the pharmaceutical company.
BeSD domains covered	Thinking and Feeling<br>Motivation<br>Practical Issues
Considerations in the context of the current study	This study does not identify nor compare types of communication tools that influence horse owner vaccine uptake. It provides descriptive information on horse owner desires in communication around HeVD and identifies shortfalls of HeVD communication.

**The Hendra virus vaccine: Perceptions regarding the role of antibody titre testing**<br>Barrett, RS, Wiethoelter, A, Halpin, K<br>Australian Veterinary Journal, 2021	
Population	Veterinarians who had submitted HeV-vaccinated horse serum samples to the Australian Centre for Disease Preparedness (ACDP) laboratory for HeV vaccination antibody titre testing.
Equine sectors represented	Commercial enterprise i.e., veterinary/equine health services
Data collection period	Not reported.
Sample size	N = 6
Study type	Qualitative study
Data collection tool	Semi-structured telephone interviews.<br>Each interview consisted of 8 open-ended questions covering 3 main topics: (1) personal experience with HeV vaccination antibody titre testing, (2) practice policies around HeV vaccination and titre testing, and (3) communication of titre test results with horse owners.
Analytic approach	Audio recordings of each telephone interview were transcribed for thematic analysis.

Objectives	Explain horse owners' and veterinarians' perception of the application and understanding of HeV antibody titre testing and how the test result influences veterinary advice.<br>Gain insight into the relationship between veterinarians and horse owners and the communication between the two parties regarding HeV antibody titre testing.
Factors studied	Experiences, knowledge, perceptions, practices, and communication of HeV vaccination antibody titre testing.
Main findings	HeV vaccination antibody titre testing was reported as an alternative to HeV vaccination by horse owners.<br>Fear of vaccine reactions and over-vaccination were also reported as triggers for HeV vaccination antibody titre testing.<br>Most veterinarians reported difficulty in communicating the results of the titre tests to owners, the titre cut-off value, and the relationship between the titre value and protective immunity.<br>Veterinarians used verbal discussion (telephone or in-person) and provision of a copy of the test results (email or a hard-copy given in-person) when communicating the titre test result to clients.<br>Client understanding/comprehension was perceived as low by most of the veterinarians regardless of the implementation of a multimodal communication approach.<br>One veterinarian who claimed no difficulty in communicating titre test results to clients or client understanding had a background in immunology/science prior to commencing their veterinary studies.
BeSD domains covered	Thinking and Feeling<br>Motivation<br>Practical Issues
Considerations in the context of the current study	This study does not compare types of communication tools that influence vaccine uptake behaviour. It provides descriptive information on the uptake of vaccination alternatives, the use of a multimodal approach to client communication, and the advantage of a strong scientific background to client communication.
